# Acute Decompensated Valvular Disease in the Intensive Care Unit

**DOI:** 10.1016/j.jacadv.2024.101402

**Published:** 2024-12-26

**Authors:** P. Elliott Miller, Balimkiz C. Senman, Ann Gage, Anthony P. Carnicelli, Mark Jacobs, Aniket S. Rali, Mourad H. Senussi, Ankeet S. Bhatt, Steven M. Hollenberg, Annapoorna Kini, Venu Menon, Kendra J. Grubb, David A. Morrow, American College of Cardiology Critical Care Cardiology Section

**Affiliations:** a Section of Cardiovascular Medicine, Yale School of Medicine, New Haven, Connecticut, USA; b Division of Cardiology, Duke University, Durham, North Carolina, USA; c Centennial Heart, Centennial Medical Center, Nashville, Tennessee, USA; d Division of Cardiology, Department of Medicine, Medical University of South Carolina, Charleston, South Carolina, USA; e Division of Cardiology, Stony Brook University, Stony Brook, New York, USA; f Division of Cardiovascular Diseases, Vanderbilt University Medical Center, Nashville, Tennessee, USA; g Department of Anesthesiology, Vanderbilt University Medical Center, Nashville, Tennessee, USA; h Department of Cardiology, Baylor College of Medicine, Houston, Texas, USA; i Texas Heart Institute, Houston, Texas, USA; j Kaiser Permanente San Francisco Medical Center and Division of Research, San Francisco, California, USA; k Division of Cardiovascular Medicine, Stanford School of Medicine, Palo Alto, California, USA; l Emory Heart & Vascular Institute, Emory University School of Medicine, Atlanta, Georgia, USA; m Division of Cardiology, Mount Sinai Hospital and Icahn School of Medicine at Mount Sinai, New York, New York, USA; n Department of Cardiovascular Medicine, Heart and Vascular Institute, Cleveland Clinic, Cleveland, Ohio, USA; o Division of Cardiothoracic Surgery, Emory University School of Medicine, Atlanta, Georgia, USA; p TIMI Study Group, Cardiovascular Division, Brigham and Women’s Hospital, Harvard Medical School, Boston, Massachusetts, USA

**Keywords:** cardiac intensive care unit, cardiogenic shock, valvular disease

## Abstract

Acute decompensated valvular disease encompasses a group of complex and challenging conditions, which are often the primary reason for admission to the cardiac intensive care unit and can also complicate the management of other primary cardiac disorders. Critically ill patients with valvular disease also present unique diagnostic and management challenges. Historically, medical and percutaneous interventional therapies have been limited and surgery was the only definitive treatment; however, surgical risk can at times be prohibitive. High-quality evidence to direct management of acute valvular disorders in this population is lacking and societal guidelines largely do not address treatment options for critically ill patients with decompensated valvular disease. In this review, we discuss the clinical presentation and epidemiology of commonly encountered valvular diseases in the modern cardiac intensive care unit, highlight key pathophysiology, detail gaps in evidence, describe the pivotal role of multidisciplinary Heart Teams, and provide guidance for management.

## Highlights

• Patients with decompensated valvular disease in the ICU are critically ill and complex.
• Decompensated valvular disease requires a multidisciplinary team and tailored therapies.
• Future valvular trials including critically ill patients are needed to improve the evidence base.

Acute decompensated valvular disease encompasses a group of complex and challenging conditions, which are often the primary reason for admission to the cardiac intensive care unit (CICU) and can also complicate the management of other primary cardiac disorders. Data from tertiary care CICUs in North America demonstrate that approximately 8% of patients are admitted to the CICU for a primary valvular etiology, and 14% have severe valvular disease.^1^ Furthermore, the increasing comorbidity of the CICU patient population has complicated the management of decompensated valvular disease.^2^ Compared to patients without valvular disease, patients with valvular disease complicating cardiogenic shock have substantially higher in-hospital mortality.^3^, ^4^, ^5^, ^6^

Historically, medical and percutaneous interventional therapies have been limited and surgery was the only definitive treatment; however, surgical risk can at times be prohibitive and its assessment often varies between institutions and surgeons. Balloon aortic valvuloplasty (BAV) can be used as a temporizing option with low rates of procedural complications and limited contrast administration; long-term outcomes are poor with the majority having recurrence of severe stenosis after 6 to 12 months.^7^^,^^8^ Although tested in elective settings, improvement in transcatheter technologies has expanded the urgent and emergent therapeutic options for valvular heart disease with prohibitive surgical risk. Randomized clinical trials of transcatheter interventions for many valvular pathologies have excluded or poorly represented patients with acute critical illness (Table 1). Similarly, clinical trials assessing common CICU diagnoses, such as cardiogenic shock, commonly exclude patients with severe valvular disease.^9^ Accordingly, high-quality evidence to direct management of acute valvular disorders in this population is lacking and societal guidelines largely do not address treatment options for critically ill patients with decompensated valvular disease.^10^^,^^11^ Critically ill patients also present unique challenges as some diagnostic tests may not be feasible for patients in the CICU.

**Table 1:** Pivotal Randomized Controlled Trials for the Percutaneous Management of Severe Valvular Disease

Clinical Trial	Year	N	Comparison	Critical Care-Related Exclusion Criteria	Primary Outcome
PARTNER B	2010	358	Nonsurgical candidates randomized to TAVR vs medical therapy	• AMI within 30 d • Vasoactive or mechanical support • CAD requiring revascularization • LVEF <20% • TIA/stroke within 6 mo • Creatinine >3.0 mg/dL or ESRD • Any blood dyscrasia	Lower mortality with TAVR at 1 y
PARTNER A	2011	699	High-risk patients randomized to TAVR vs SAVR	Same as PARTNER B	TAVR noninferior for mortality at 1 y
US CoreValve High-Risk Study	2014	795	Increased surgical risk randomized to TAVR vs SAVR	• AMI within 30 d • Untreated significant CAD • Cardiogenic shock requiring inotropic or mechanical support • LVEF <20% • TIA/stroke within 6 mo • ESRD • Any blood dyscrasia	Lower mortality with TAVR at 1 y
PARTNER 2	2016	2,032	Intermediate-risk patients randomized to TAVR vs SAVR	• AMI within the last 30 d • BAV within the last 30 d • LVEF <20% • Complex CAD • TIA/stroke within 6 mo • Creatinine >3.0 mg/dL or ESRD • Any blood dyscrasia	TAVR noninferior for all-cause mortality or disabling stroke at 2 y
SURTAVI	2017	1,746	Intermediate-risk patients randomized to TAVR vs SAVR	• AMI within 30 d • Contraindication to ECMO • Cardiogenic shock • LVEF <20% • Uncontrolled atrial fibrillation • TIA/stroke within the 6 mo • Child-C liver failure • ESRD • Severe COPD • Blood dyscrasias	TAVR noninferior for all-cause mortality or disabling stroke at 2 y
COAPT	2018	614	MitraClip vs medical therapy for severe mitral regurgitation	• COPD requiring home oxygen • TIA/stroke within 30 d • Stage D heart failure • Hemodynamic instability requiring inotropic or mechanical support	Lower rate of hospitalization for heart failure with MitralClip
PARTNER 3	2019	1,000	Low-risk patients randomized to TAVR vs SAVR	• AMI within 30 d • Inotropic or mechanical support within 30 d • Mechanical ventilation within 30 d • LVEF <30% • Complex CAD • TIA/stroke within 90 d • Severe lung disease or pHTN • Any active liver disease • Any blood dyscrasia	Lower composite outcome of death, stroke, or rehospitalization for TAVR at 1 y
Evolut Low Risk	2019	1,468	Low-risk patients randomized to TAVR vs SAVR	• AMI within 30 d • Cardiogenic shock • TIA/stroke within 2 mo • Blood dyscrasias	TAVR noninferior for the composite outcome of death or disabling stroke at 2 y
UK-TAVI	2022	913	Intermediate-risk patients randomized to TAVR vs SAVR	N/A	TAVR noninferior for all-cause mortality at 1 y

AMI = acute myocardial infarction; BAV = balloon aortic valvuloplasty; CAD = coronary artery disease; COPD = chronic obstructive lung disease; ECMO = extracorporeal membrane oxygenation; ESRD = end-stage renal disease; LVEF = left ventricular ejection fraction; pHTN = pulmonary hypertension; SAVR = surgical aortic valve replacement; TAVR = transcatheter aortic valve replacement; TIA = transient ischemic attack.

In this State-of-the-Art review, we discuss the clinical presentation and epidemiology of commonly encountered valvular diseases in the modern CICU, highlight key pathophysiology, detail gaps in evidence, describe the pivotal role of multidisciplinary Heart Teams, and provide guidance for management (Central Illustration).

**Central Illustration:**
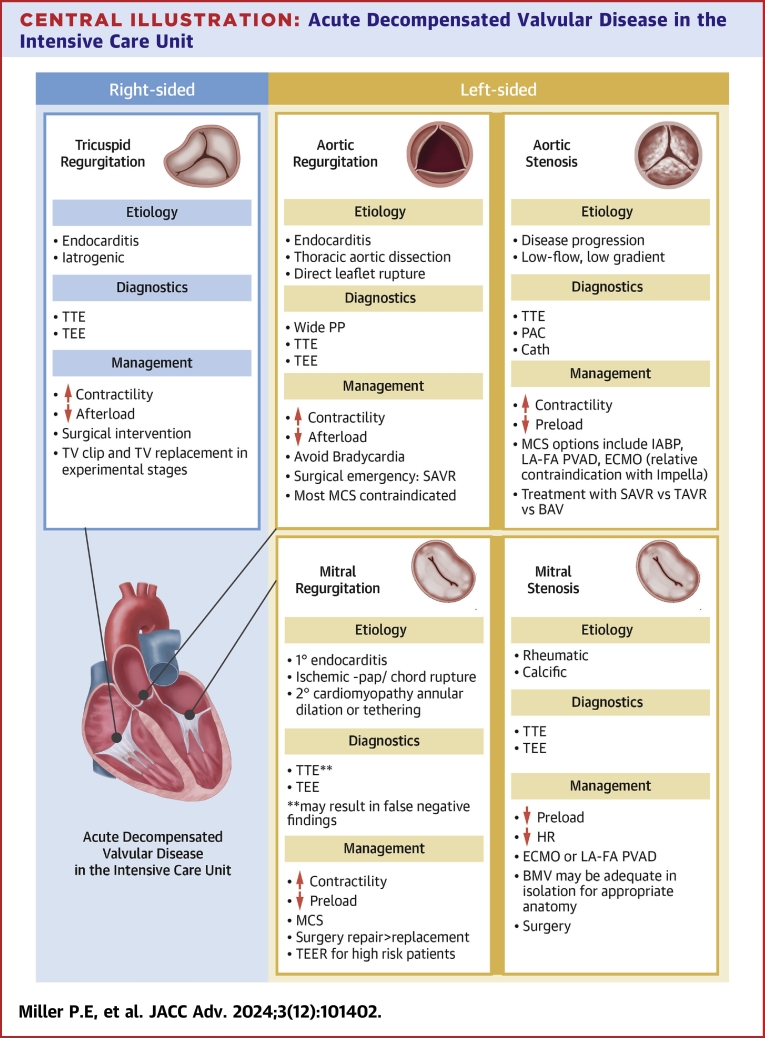
**Acute Decompensated Valvular Disease in the Intensive Care Unit** Etiology, diagnostic, and management for right- and left-sided valvular disease. MS = mitral stenosis; PP = pulse pressure; TV = tricuspid valve; other abbreviations as in Figure 2, Figure 3, Figure 4 and 6.

## Initial approach to decompensated valvular disease

There are many similarities to the initial diagnostic approach and evaluation of patients across the spectrum of decompensated valvular disease. A comprehensive assessment should begin with a thorough physical examination, with the important caveat that acute murmurs may be soft or “silent” due to early equalization of pressures.^12^ The most readily available and useful imaging modality includes point-of-care or formal transthoracic echocardiography (TTE). Not uncommonly in the ICU, transthoracic image quality may not be ideal in the acute setting due to poor windows, equalization of pressures, or eccentric regurgitant jets, making transesophageal echocardiography (TEE) often critical for defining severity and for procedural planning.^13^ Cardiac computed tomography (CT) is an alternative diagnostic technique. However, reasonable hemodynamic stability is necessary to accommodate obtaining the study and the imaging is typically performed using intravenous contrast, which may be detrimental to patients with cardiogenic shock and kidney injury. Furthermore, irregular or elevated heart rates, common in patients with decompensated valvular disease, may impact image quality.

Once a decompensated patient is found to have severe valvular disease, initial management should be guided by a thorough volume assessment, including both physical examination and objective measure of filling pressures, ideally with a pulmonary artery catheter (PAC). In an analysis from the Cardiogenic Shock Working Group, complete hemodynamic profiling using a PAC in patients with cardiogenic shock was associated with lower in-hospital mortality compared to incomplete profiling or absence of hemodynamic assessment.^14^ Notably, 15% (214/1,025) of patients had documented valvular heart disease (unspecified valve and severity) and the majority of these patients underwent either complete (64%) or partial (26%) hemodynamic assessment. While the investigators did not assess for effect modification by presence or absence of valvular disease, these findings indicate that invasive hemodynamic profiling is commonly pursued in this population. Ultimately, hemodynamic tailoring with diuretics, vasoactive medications, and mechanical circulatory support (MCS) should be guided by both objective measures from the PAC, including a central venous pressure target between 5 and 10 mm Hg, pulmonary artery wedge pressure generally ≤15 mm Hg, and cardiac index ≥2.2 L/min/m^2^, as well as markers of appropriate perfusion, such as lactate, mental status, and urine output.

**Figure 1:**
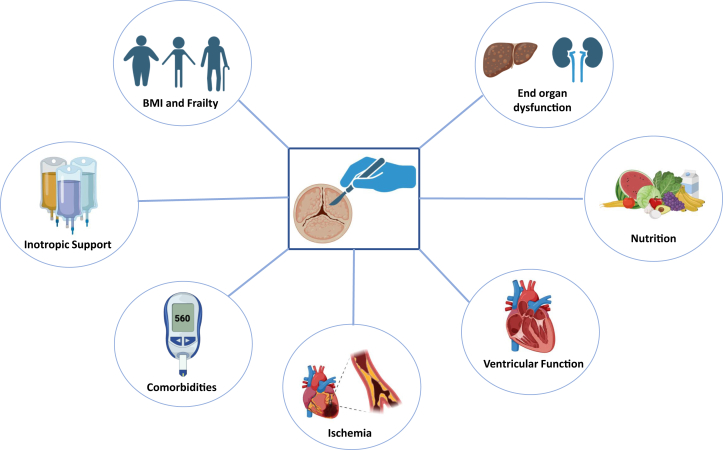
**Patient-Specific Factors Influencing Surgical Decisions** Created in BioRender (2024). AV = aortic valve; BMI = body mass index; BP = blood pressure; CABG = coronary artery bypass grafting; LAP = left atrial pressure; LVEDV = left ventricular end-diastolic volume; NE = norepinephrine; SVR = systemic vascular resistance.

Treatment decisions should involve a multidisciplinary discussion including a cardiac imaging specialist, structural interventionalist, and cardiac surgeon. Definitive procedural or surgical candidacy includes a complex interplay of factors that may be different from patient-to-patient, valve pathology, and ultimately from surgeon-to-surgeon (Figure 1). While often challenging, it is important to try to differentiate whether the severe valvular disease is the primary inciting pathology or a contributor to decompensation. Clinicians should conduct a careful history, review of previous records, and utilize imaging to assess for the mechanism of valve dysfunction as well as for signs of chronic remodeling. Acute valvular dysfunction is more likely to be the etiology of decompensation and may need to be directly addressed early in the clinical course. Alternatively, chronic valvular dysfunction can certainly be the cause of decompensation but often allows more time for medical stabilization and procedural planning. If an intervention is considered, a full assessment of the options, patient comorbidities, and risk of both morbidity and mortality is necessary. Lastly, and most importantly, the ultimate procedural decision should include shared decision-making with the patient and their family.

## Specifics by valvular pathology

### Aortic stenosis

#### Epidemiology

Aortic stenosis (AS) is one of the most commonly encountered valvular pathologies, with severe disease present in 3.4% of individuals >75 years of age in the United States.^15^ In patients with left ventricular (LV) dysfunction, severe AS can significantly reduce cardiac output and is associated with an extremely high mortality in patients who progress to cardiogenic shock.^16^ While cardiogenic shock is a common diagnosis among patients admitted to contemporary CICUs, prevalent in approximately 15% of total CICU admissions,^17^ the portion of these patients with underlying severe AS is poorly described.

#### Diagnosis and evaluation

**Figure 2:**
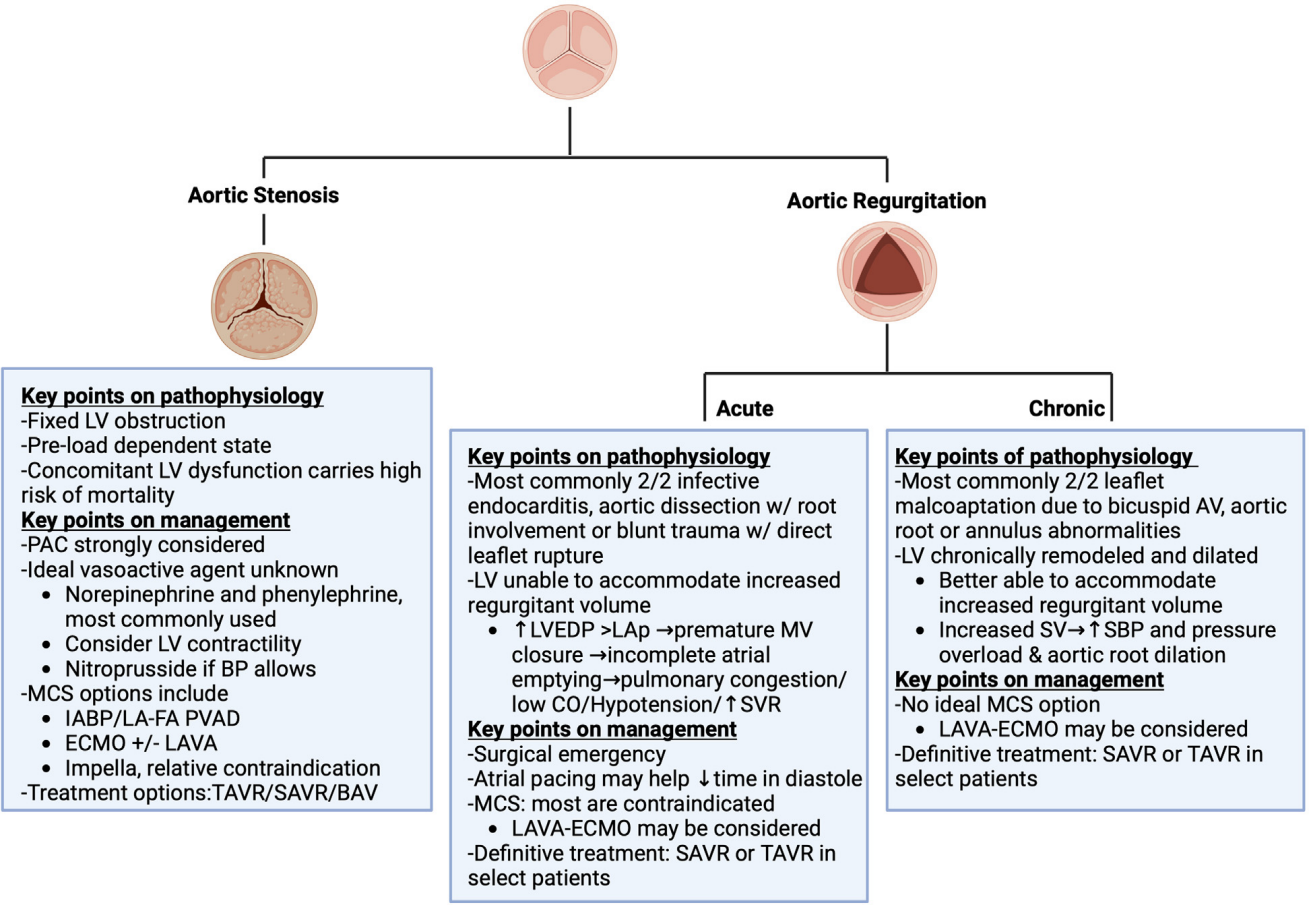
**Key Points for Aortic Stenosis and Regurgitation** Created in BioRender (2024). BAV = balloon aortic valvuloplasty; CO = cardiac output; ECMO = extracorporeal membrane oxygenation; IABP = intra-aortic balloon pump; LA-FA PVAD = left-atrial to femoral-artery percutaneous ventricular assist device; LAVA = left atrial-veno-arterial; LV = left ventricle; LVEDP = left ventricular end-diastolic pressure; MCS = mechanical circulatory support; MV = mitral valve; PAC = pulmonary artery catheter; SAVR = surgical aortic valve replacement; SBP = systolic blood pressure; SV = stroke volume; TAVR = transcatheter aortic valve replacement.

In patients without a diagnosis of severe AS, establishing this diagnosis in the decompensated patient can be challenging. The most used and readily available modality at the bedside is TTE (Figure 2). However, Doppler measurements rely on sufficient cardiac output across the stenotic valve to generate diagnostic flow velocities.^13^ Cardiac output in patients with cardiogenic shock is severely reduced, negatively affecting the sensitivity of echocardiography for the diagnosis of severe AS. Given these difficulties, diagnosing true severe AS from low-flow, low-gradient AS in the decompensated patient can be challenging without the ability to conduct common testing, such as dobutamine stress echocardiography. In addition to those with low-flow, low-gradient severe AS, a stroke volume index ≤35 ml/m^2^ can help differentiate low- vs normal-flow low-gradient AS in those with preserved ejection fraction.^10^ Cardiac CT is an alternative diagnostic technique if feasible based on the acuity of illness. Furthermore, irregular or elevated heart rates, common in patients with decompensated valvular disease, may impact image quality. Ideally, heart rates should be <70 beats/min, although higher heart rates may be acceptable depending on the type of CT scanner. Invasive valve assessment is subject to the same transvalvular flow limitations that render echocardiography less accurate in patients with low cardiac output.^18^

The presence of severe AS introduces several potential pitfalls in interpreting conventional, noninvasive intravascular volume and cardiac output measures. In patients with cardiogenic shock or decompensated heart failure with severe AS, obtaining a complete hemodynamic profile via a PAC may provide useful diagnostic information to tailor diuretic and hemodynamic support therapies.

#### Management

The initial management of the decompensated patient with severe AS will commonly require a careful decongestive strategy. Patients with AS are preload sensitive, due to the fixed obstruction at the aortic valve, and over-diuresis may lead to hemodynamic collapse. Therefore, a careful volume assessment is essential as the septic patient with AS will require a substantially different strategy than the AS patient with decompensated heart failure. For the congested patient, the initial diuretic regimen should not be significantly different than those without severe AS. However, whenever there is uncertainty regarding the adequacy of preload or signs of shock, we favor placement of a central venous catheter or PAC for ongoing management generally targeting a central venous pressure between 6 and 10 mm Hg. Cardiac output monitoring via a PAC also may allow for a more controlled titration of vasoactive medications to stabilize or bridge to definitive therapies.

The selection of vasoactive medications in severe AS is poorly studied and not addressed in societal guidelines.^10^^,^^11^ Medications with predominantly inotropic properties (eg, dobutamine) may increase the gradient across the fixed stenosis, worsen myocardial ischemia if present, and/or provoke atrial or ventricular arrhythmias, all of which are poorly tolerated in an acutely decompensated patient with valvular disease. Norepinephrine and phenylephrine are the most prescribed vasopressors, but with limited evidence. Both medications increase mean arterial pressure and ideally improve coronary perfusion pressure. However, the supporting evidence is comprised of 2 small, decades-old studies.^19^, ^20^, ^21^ Patients with mixed shock and/or LV dysfunction may benefit from the beta-adrenergic effects of norepinephrine while those with preserved systolic function may respond well to phenylephrine, which supports the mean arterial pressure without the risk of precipitating arrhythmias. In patients who are not hypotensive, nitroprusside is an option. In a small study (n = 25) of patients with systolic heart failure, mean cardiac index of 1.6 L/min^2^, and severe AS, nitroprusside increased the cardiac index to 2.2 L/min/m^2^ and 2.5 L/min/m^2^ at 6 and 24 hours, respectively.^22^ Notably, patients requiring a vasopressor or with a mean arterial pressure ≤60 mm Hg were excluded. Since AS is a preload-sensitive state, vasodilators which primarily reduce preload, such as nitroglycerin, should be used with caution or avoided.

**Figure 3:**
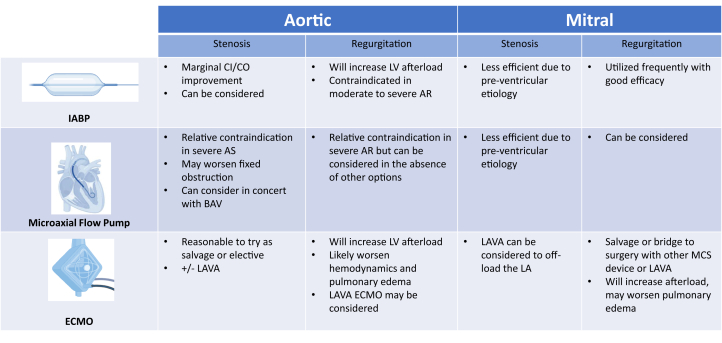
**Temporary Mechanical Circulatory Support Treatment of Valvular Disease** Created in BioRender (2024). AR = aortic regurgitation; AS = aortic stenosis; CI = cardiac index; LA = left atrium; other abbreviations as in Figure 2.

In patients unresponsive to vasoactive medications, temporary MCS may be considered (Figure 3). The primary goals of MCS (with or without AS) are to unload the left heart and improve systemic perfusion. These goals may be difficult to achieve in the setting of severe AS. Intra-aortic balloon pump (IABP) counter-pulsation offers marginal unloading and modest cardiac output augmentation even without fixed LV obstruction. Furthermore, it should be avoided in patients with significant aortic regurgitation (AR). In a small case series (n = 25), including patients with AS (mean valve area 0.64 cm^2^) and cardiogenic shock (mean cardiac index 1.77 L/min/m^2^), mean cardiac index increased to 2.18 L/min/m^2^ and 2.36 L/min/m^2^ at 6 and 24 hours, respectively (both, *P* < 0.001).^23^ Severe AS has been considered a relative contradiction for transvalvular microaxial percutaneous ventricular assist devices (Impella, Abiomed Inc).^24^ Nevertheless, several single-center and one registry report the feasibility of Impella use, sometimes preceded by a BAV, in patients with AS and cardiogenic shock.^25^, ^26^, ^27^ Although feasible, caution should be taken as the large bore device can worsen outflow obstruction.^28^ MCS using transseptal cannulation of the left atrium and an external ventricular assist device (eg the previously available TandemHeart pump, LivaNova, or CentriMag, Abbott) has offered another percutaneous option. However, only small case reports and series describe the use of transseptal cannulation for support of patients with AS and cardiogenic shock.^29^^,^^30^ This approach requires specialized experience with transseptal delivery and carries the risk of iatrogenic atrial septal defect creation.^31^ Finally, extracorporeal membrane oxygenation (ECMO) has been used both electively and as a salvage therapy for shock complicating aortic interventions. Additional considerations when deploying ECMO for any etiology is whether LV unloading through other MCS devices or left atrial veno-arterial cannulation (so-called “LAVA”) is warranted. Through transseptal catheter drainage of the left and right atria, LAVA provides an approach at ventricular unloading without a second arterial access or need to cross the aortic valve.^32^^,^^33^

**Figure 4:**
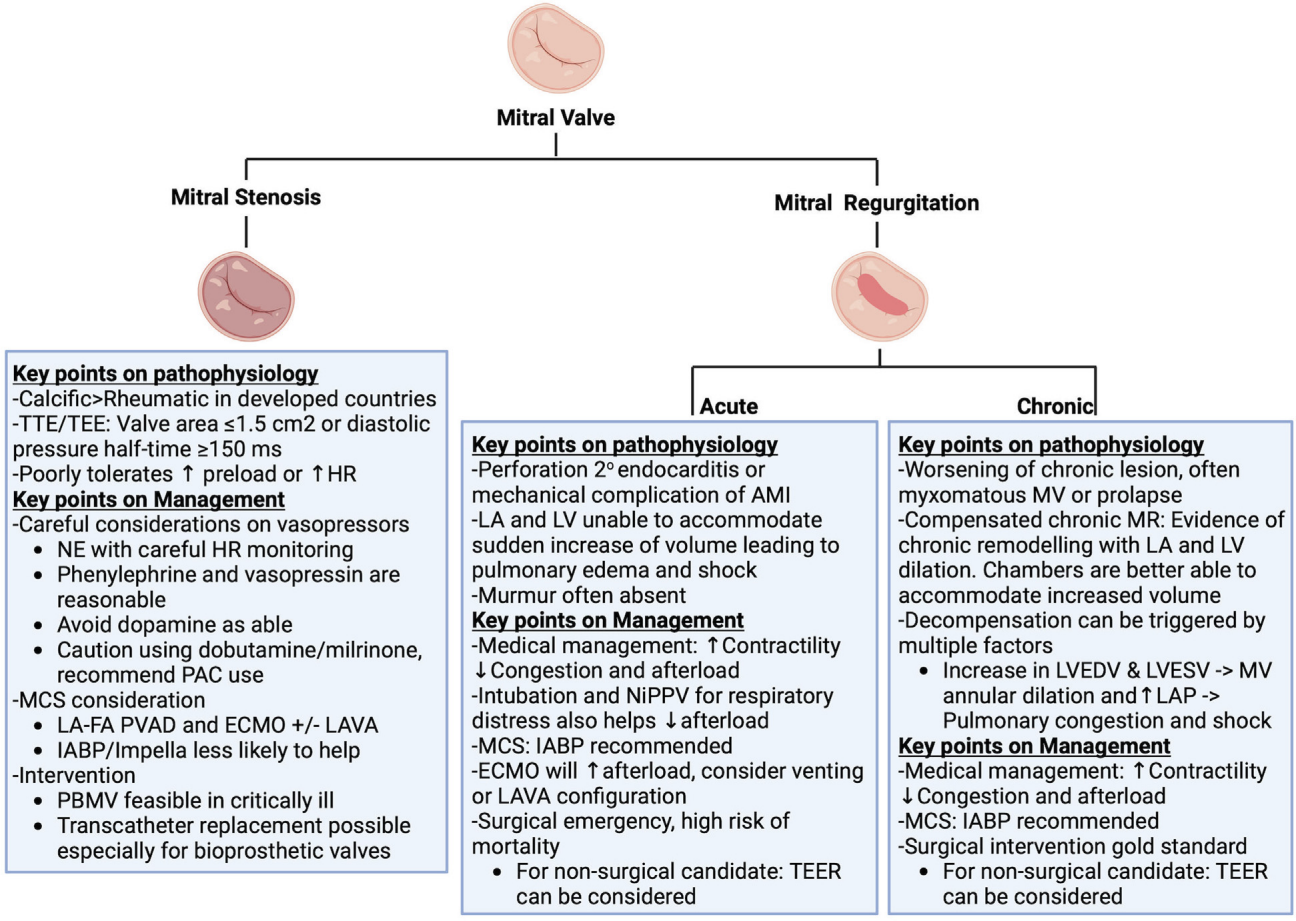
**Key Points for Mitral Stenosis and Regurgitation** Created in BioRender (2024). AMI = acute myocardial infarction; HR = heart rate; LAP = LA pressure; LVESV = LV end-systolic volume; MR = mitral regurgitation; NiPPV = noninvasive positive pressure ventilation; PBMV = percutaneous balloon mitral valvuloplasty; TEE = transesophageal echocardiogram; TEER = transcatheter edge-to-edge repair; TTE = transthoracic echocardiography; other abbreviations as in Figures 2 and 3.

Diuretics, vasoactive medications, respiratory support, and MCS are short-term therapies for optimizing the patient’s clinical status in preparation for definitive treatment. The severity of any underlying cardiomyopathy is an important factor to consider when deciding on candidacy for aortic valve intervention. A multidisciplinary approach to clinical stabilization and identifying opportunities for destination therapies is recommended. This approach should involve members of the multidisciplinary shock team (critical care cardiologist, advanced heart failure cardiologist, cardiac surgeon, interventional cardiologist), members of the multidisciplinary valve team (structural interventionalist, cardiac imaging specialist), and palliative care. Surgical valve replacement in the acute setting is often not offered to patients in cardiogenic shock due to an extremely high peri-operative mortality rate.^34^ BAV may be a feasible “rescue” therapy for patients with severe AS and cardiogenic shock, performed alone or toward staged more definitive interventions.^35^ However, in-hospital and long-term mortality in this population remains high. One case series of 40 patients with AS and cardiogenic shock or refractory pulmonary edema demonstrated a 30% early mortality after BAV.^36^^,^^37^

To date, patients with cardiogenic shock have been excluded from randomized controlled trials assessing transcatheter aortic valve interventions (Table 1). A large real-world analysis from the Society of Thoracic Surgeons (STS)/American College of Cardiology (ACC) Transcatheter Valve Therapy Registry compared the epidemiology and outcomes of patients undergoing aortic valve intervention with and without preprocedural cardiogenic shock.^38^ Among ∼300,000 patients from 2015 to 2022, 5,006 had preprocedural cardiogenic shock before balloon-expandable transcatheter aortic valve replacement (TAVR). At 30 days, mortality was higher in patients with cardiogenic shock (12.9% vs 4.9%). However, in landmark analysis for patients that survived to 30 days, 1-year mortality was similar between those with and without preprocedural cardiogenic shock.^38^ Historical benchmarks for quality are unlikely to apply in this population as existing metrics were developed without significant numbers of patients with cardiogenic shock. An alternative strategy may involve BAV for initial relief of the LV obstruction followed by medical management of cardiogenic shock and eventually TAVR pending resolution of shock physiology; although this strategy exposes the patient to the risks of 2 procedures. There are insufficient data to guide the selection between these strategies.

### Aortic regurgitation

#### Epidemiology and pathobiology

Although less common than AS,^39^ decompensated AR can occur both acutely and chronically, with substantially different pathophysiology and treatments (Figure 2).^40^ Several etiologies of acute severe AR exist, with infective endocarditis and thoracic aortic dissection involving the aortic root being the most common. Blunt chest trauma may also lead to severe AR from aortic dissection or direct leaflet rupture.^41^ Etiologies of chronic AR include both malcoaptation of the aortic valve due to a congenitally bicuspid valve or rheumatic heart disease, as well as abnormalities of the aortic root or annulus, such as familial aortopathy and connective tissue syndromes.^40^ Due to the acute drop in effective cardiac output and significant elevation in LV filling pressures, patients with acute AR often present in a state of hemodynamic instability and pulmonary edema.

When severe AR occurs acutely, the LV is poorly equipped to maintain sufficient forward stroke volume due to the large regurgitant fraction. This condition results in LV end-diastolic pressure exceeding that of the left atrium, premature closure of the mitral valve during diastole, and incomplete atrial emptying. Increased left atrial pressure and decreased LV filling result in increased pulmonary pressures, respiratory failure, low cardiac output, hypotension, and compensatory increased systemic vascular resistance. In contrast, in chronic AR, the LV has had the opportunity to dilate and remodel to accommodate the excess LV end-systolic volume.^40^ Systolic hypertension and pressure overload develop from the increase in total stroke volume, including both forward and regurgitant volume,^42^ which further dilates the aortic root.

#### Diagnosis and evaluation

Timely recognition of acute severe AR is critical to altering the natural progression of this disease and should prompt a thorough physical examination and echocardiographic evaluation. Importantly, the well-recognized physical examination findings of chronic AR, such as widened pulse pressure, may not be present. In fact, the pulse pressure is usually narrow or normal in acute severe AR due to the rapid equalization of pressures between the LV and aorta.

#### Management

Acute severe AR is a surgical emergency. Medical management focuses on stabilizing the patient in preparation for surgical intervention. However, specific professional society recommendations on medical therapies are lacking.^10^^,^^11^ For both decompensated chronic and acute severe AR, treatments should be tailored toward afterload reduction and decongestion. For hypertensive or normotensive patients, a pure vasodilator like nitroprusside is considered a first-line therapy. For hypotensive patients, the judicious use of vasoconstrictors and inotropes, ideally with PAC monitoring, is necessary. Purely vasoconstrictive agents such as phenylephrine and vasopressin may worsen AR while agents with inotropic properties, such as norepinephrine and epinephrine, may be better tolerated. Chronic AR is associated with a wide pulse pressure which often leads to a lower diastolic blood pressure. Blood pressure targets will need to be individualized, and clinicians should be aware of the possibility of precipitating coronary ischemia with very low diastolic blood pressure in this setting.

In patients with aortic dissection as a cause of acute AR, while beta-blocker therapy and heart rate control are important to reduce shear stress, prolonged diastole will worsen acute AR and may lead to further decompensation. Furthermore, higher heart rates reduce diastolic filling times, reducing the regurgitant volume and thereby LV end-diastolic volumes.^43^ In patients without aortic dissection, targeting a faster heart rate, via atrial pacing, has been proposed as a therapy to reduce AR temporarily.^44^ MCS options are limited or contraindicated in patients with AR (Figure 3). Both IABP counter-pulsation and peripheral VA-ECMO will increase afterload and likely worsen hemodynamics and pulmonary edema. Severe AR has similarly been considered a contraindication to use of Impella. LAVA-ECMO may offer an option for patients with cardiogenic shock and AR in experienced centers.^45^ Ultimately, surgical replacement is the standard treatment. Rescue TAVR has been shown to be feasible in patients with refractory CS, but the published experience is limited to small case series.^46^^,^^47^

### Mitral regurgitation

#### Epidemiology and pathobiology

Acute mitral regurgitation (MR) or sudden worsening of pre-existent MR are among the most common valvular pathologies that precipitate or complicate existing shock.^48^ Primary MR is defined as an abnormality in the MV apparatus and is commonly due to myxomatous degeneration and prolapse of the mitral valve leaflets. However, several additional pathologies, including leaflet perforation (eg, from endocarditis) and mechanical complications of acute myocardial infarction may lead to abrupt, severe MR and resultant cardiogenic shock.^12^ In particular, acute papillary muscle rupture, often in the setting of acute myocardial infarction, is a surgical emergency. The incidence of such rupture has decreased drastically, primarily driven by improvements in early reperfusion.^49^ Presentation of patients with acute ischemic MR can range from mild dyspnea to cardiogenic shock, though mild presentations can precipitously deteriorate to severe hemodynamic compromise, especially with papillary muscle rupture (Figure 4).^50^ Mortality associated with this diagnosis remains high, reaching approximately 80% in patients who are unable to undergo surgical correction and 19% to 53% with operative intervention.^48^^,^^51^

In contrast, secondary MR occurs due to the impaired ability of structurally normally MV leaflets to coapt properly. It is important to note that secondary MR is particularly dynamic condition depending on loading conditions, including volume status, blood pressure, and vasoactive medication use.^52^ The most common cause of secondary MR is ventricular remodeling subsequent to pressure/volume overload, seen in both ischemic and nonischemic etiologies. There is growing recognition that atrial myopathy and resultant atrial dilation may similarly contribute to secondary MR, termed atrial functional MR. Atrial functional MR can be acutely worsened during conditions causing an excess load on the left atrium or impaired normal left atrial contractility, such as atrial fibrillation.^53^

#### Diagnosis and evaluation

The murmur of acute MR may be soft or even absent, and presentations are frequently associated with pulmonary edema.^12^ Chest radiographs commonly show bilateral pulmonary edema but may also present with unilateral pulmonary edema (typically right-sided) due to an eccentrically directed jet and can be confused with other etiologies such as unilateral pneumonia.^54^ MR may be particularly prone to underdiagnosis of severity through TTE due to jet eccentricity. If significant MR is suspected but the severity is unclear after transthoracic imaging, a TEE should be obtained. Beyond investigation with echocardiography, significant MR should be suspected in those with a PAC that have large V waves.

#### Management

Treatment will vary by presentation, mechanism of MR (eg, primary vs secondary), anatomy of the mitral valve, comorbid conditions, and especially the acuity of the MR. Acute management options include medical therapy, MCS, transcatheter approaches, and ideally urgent mitral valve surgery for suitable candidates. Alternatively, given the risk of causing worsened LV function with mitral valve intervention, the approach to decompensated valvular disease associated with chronic MR requires careful consideration for both procedural and medical options. Medical therapy serves to stabilize patients with primary MR before definitive therapy or improve forward flow for patients with decompensated secondary MR or those who are not surgical candidates.

In general, medical therapy with vasodilatory properties, with or without inotropy, is preferred in patients with severe MR complicating cardiogenic shock. Agents such as sodium nitroprusside, may be used to lower LV afterload, improve cardiac output, and lower regurgitant volumes, but their use may be limited by hypotension. Purely vasoconstrictive agents, such as phenylephrine, may increase LV afterload and worsen existing MR and should generally be avoided in this setting. If MR and cardiogenic shock lead to acute respiratory distress, noninvasive and invasive positive pressure ventilation (PPV) (discussed in depth below) may not only improve work of breathing but also reduce LV afterload, reduce MR, and improve forward flow.^55^^,^^56^

In cases in which hemodynamic status remains compromised despite medical therapy, MCS should be considered. Although clinical trial data are lacking for this specific population, IABP counter-pulsation has been identified as a reasonable option for patients with cardiogenic shock complicated by MR and is utilized frequently in the United States.^49^^,^^57^ Translational models suggest that IABP counter-pulsation can reduce the regurgitant fraction and subsequently improve cardiac output.^58^ In patients with acute on chronic heart failure associated cardiogenic shock, moderate to severe MR has been reported to be a predictor of a favorable hemodynamic response to IABP (so called “super-responders”).^59^ Reports with other percutaneous MCS devices are sparse,^60^^,^^61^ but Impella support is a reasonable option with special care to avoid the mitral apparatus. ECMO can be considered as a salvage therapy in refractory shock or as a bridge to surgery but will increase afterload and may potentially worsen MR and pulmonary edema. If ECMO is selected, ideally it is used with LV-venting, with either an IABP or Impella, or in the LAVA configuration.

In patients with primary MR complicated by cardiogenic shock, surgery is considered the standard treatment.^10^^,^^11^ In particular, acute papillary muscle rupture is a surgical emergency. For patients who are not surgical candidates, transcatheter edge-to-edge repair (TEER) of the mitral valve, such as with MitraClip (Abbott Structural Heart), may be considered. There are several case reports and at least one retrospective, multicenter study describing the feasibility, procedural success, and preliminary experience with the safety of TEER in patients with refractory shock who were not surgical or durable MCS candidates.^62^, ^63^, ^64^

In a patient-level, multicenter analysis (n = 141) of patients with moderate-severe/severe MR (primary and secondary) and cardiogenic shock undergoing TEER as “salvage therapy,” in-hospital and 1-year mortality was 15.6% and 42.6%, respectively.^6^ Among 3,797 patients with cardiogenic shock from the STS/ACC Transcatheter Valve Therapy Registry, 85.6% (n = 3,249) had successful TEER placement (defined as MR grade ≤2+). Mortality at 1 year was 34.6% compared to 55.5% in those without successful TEER.^65^ However, it is important to note that this use of TEER is off-label and that robust clinical trial evidence supporting TEER in patients with cardiogenic shock is not available. The actively enrolling, single-center, randomized controlled trial, Transcatheter Mitral Valve Repair for Inotrope Dependent Cardiogenic Shock (CAPITAL MINOS, NCT05298124), is evaluating the utility of TEER compared to medical therapy in patients with cardiogenic shock and MR. The enrollment target is 144 participants with a projected completion date in 2025.^66^

### Mitral stenosis

#### Epidemiology and pathobiology

Decompensated mitral stenosis (MS) is often a consequence of either the progressive evolution of a chronic lesion or an abrupt shift in patient hemodynamics that is poorly tolerated by the stenotic valve. While rheumatic disease was historically the primary etiology of MS, the use of antibiotics for rheumatic infections and an aging demographic has resulted in the rise of MS due to calcific degeneration in higher income countries.^10^ Hemodynamically, MS results in elevated left atrial pressures and compromised LV filling. Conditions that exacerbate these hemodynamic disturbances contribute to further decompensation. Noteworthy among these conditions are tachycardia, which reduces diastolic filling time, especially during pregnancy,^67^^,^^68^ which is also associated with an increase in preload. Chronic remodeling and subsequent dilation of the left atrium renders individuals with MS susceptible to atrial fibrillation. Atrial fibrillation impairs atrial filling and diminishes diastolic filling time, especially with rapid rates, further exacerbating decompensation.^21^ Consequently, the management of decompensated MS necessitates balancing rate and rhythm control, often with agents like atrioventricular nodal blockers, digoxin, amiodarone, or cardioversion, with the potential hemodynamic consequences of some of these therapies.

#### Diagnosis and evaluation

Auscultation of MS, which often requires proper maneuvers and positioning, is sometimes not possible or challenging for the CICU patient. Mitral valve stenosis is best assessed by direct planimeter imaging with an area of ≤1.5 cm^2^ or a calculated diastolic pressure half-time ≥150 ms considered indicative of severe stenosis.^10^ A Doppler transvalvular mean gradient >5 to 10 mm Hg suggests MS but is prone to error as it is dependent on heart rate and cardiac output,^10^ which are often abnormal in the CICU.

#### Management

Given the unique pathophysiology, cardiogenic shock complicated by MS can be particularly challenging to manage. Vasopressor selection should include consideration of the potential impact on heart rate. Norepinephrine is often the first line for cardiogenic shock, and while not an unreasonable selection in this patient population, it may increase the heart rate and worsen hemodynamics. Vasopressors without chronotropic properties, such as phenylephrine with or without the addition of vasopressin, are reasonable options for patients with severe MS. Dopamine and inotropes should be avoided as a first-line option in most circumstances unless there is another compelling reason. In contrast, decompensated patients with MS may also have secondary pulmonary hypertension and/or RV failure, which may require inotropic support. In these situations, careful titration of inotropic support may be needed while avoiding tachycardia that decreases ventricular filling time. In such patients, balancing these needs for management can be very challenging and may be facilitated with continuous hemodynamic monitoring with a PAC.

MCS selection can similarly be challenging (Figure 3). Typical first-line MCS choices, such as IABP or Impella, are less likely to be effective given that the cause of shock related to MS is predominantly preventricular and the LV end-diastolic pressure is often low. Support with left atrial cannulation (eg, historically TandemHeart) has been proposed as a reasonable option as it directly offloads the left atrium.^21^^,^^69^ ECMO, potentially including a LAVA configuration for centers experienced with this mode, may be considered for patients with concomitant right heart failure and/or recalcitrant hypoxia.

For select candidates, percutaneous balloon mitral valvuloplasty (PBMV) is a feasible therapy for critically ill patients, including in resource-limited areas.^70^ Preprocedurally, it is important to discuss potential complications and whether the patient is a candidate for mitral valve replacement. The development of severe MR and subsequent cardiogenic shock following PBMV occurs in approximately 7% of patients and can be difficult to predict preprocedurally based on imaging.^71^ PBMV may also offer similarly favorable outcomes during pregnancy while being feasible even in decompensated patients.^72^^,^^73^ Transcatheter mitral valve replacement, mainly in those with stenotic bioprosthetic valves, has been reported as emergent/salvage therapy in patients with cardiogenic shock. Transcatheter mitral valve replacement is an emerging technology with unique considerations and challenges, including careful patient selection in order to avoid LV outflow tract obstruction.^74^

### Right-sided valvular disease

While isolated, acute right-sided valvular disease is less common and rarely causes cardiogenic shock,^75^ concomitant right-sided valvular disease frequently complicates left-sided disease.^76^ Etiologies causing acute tricuspid regurgitation (TR) include trauma, endocarditis (discussed below), and following device implantations. Acute traumatic TR from blunt trauma is usually tolerated well but can present with cardiogenic shock. Urgent surgical repair or replacement is rarely needed but the ideal timing is unknown.^77^ Medical management is aimed at mitigating RV volume overload as well as supporting RV contractility. There are limited available data describing percutaneous right-sided MCS devices for patients with severe TR and RV failure. Transcatheter strategies for severe TR remain in the nascent phase. There are several reports on the feasibility and success of off-label TEER for severe TR and end-stage heart failure.^78^ More recently, devices have been developed explicitly for the tricuspid valve, but evidence of their use in the acute setting is limited.

## Special scenarios

### Complicated endocarditis

**Figure 5:**
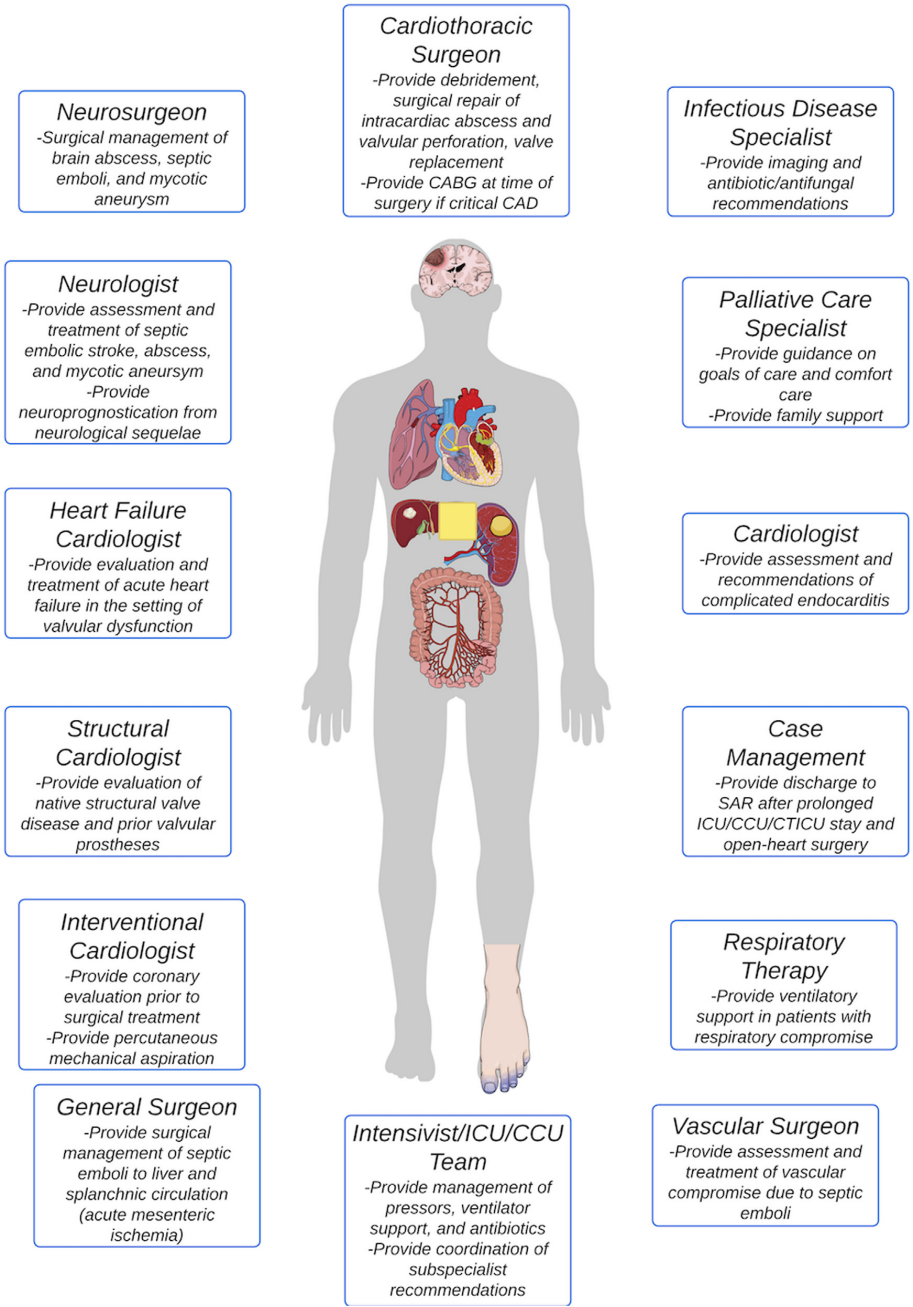
**Approach to Complicated Endocarditis in the CICU** CAD = coronary artery disease; CICU = cardiac intensive care unit; CTICU = cardiothoracic intensive care unit; ICU = intensive care unit; SAR = subacute rehab.

Mortality rates from endocarditis differ significantly based on the organism, patient demographics and comorbidities, native versus prosthetic valve, site (left- versus right-sided), and acuity. Patients with endocarditis admitted to the CICU are often critically ill with hemodynamically significant valvular disease, have complications from embolic disease including stroke, and present with mixed shock. The mortality rate for this population exceeds 50%.^79^ The complex nature of infectious endocarditis, which carries the possibility of affecting multiple organ systems simultaneously, requires a multidisciplinary approach to reduce the time to definitive treatment, improve the treatment of complications, and reduce mortality (Figure 5).^80^^,^^81^

Outcomes for critically ill patients with endocarditis in the CICU often revolve around the timing and candidacy for surgery. Indications for surgery have been well delineated,^82^ but these recommendations are often confounded in the CICU by significant comorbidity and infectious complications necessitating nuanced decision-making. Although some studies reported a mortality benefit for early surgery in patients with native valve endocarditis, the risk of selection bias remains.^83^^,^^84^ Upward of 24% of patients with a guideline indication for surgery do not go to the operating room. Given the risk of hemorrhagic conversion during cardiopulmonary bypass, the presence of stroke complicates the decision and timing of surgery.^85^ European Society of Cardiology guidelines detail recommendations on the timing of surgery in various scenarios (eg, recent stroke, hemodynamic compromise, uncontrolled infection).^86^ In addition to multidisciplinary discussions, these scenarios require comprehensive benefit and risk discussions with the patient and family.

Given limitations with imaging and reoperative risk, patients with prosthetic valve endocarditis present diagnostic and management challenges, especially among patients with critical illness in the CICU. The sensitivity of transthoracic echocardiography drops from 50% to 90% in native valves to 36% to 69% in those with prosthetic valves.^10^ TEE becomes especially important for both diagnosis (sensitivity >85%) and operative planning in such patients.^87^ For patients sufficiently stable, additional imaging with 18-fluorodeoxyglucose positron emission tomography combined with CT can improve diagnostic sensitivity and specificity (87% and 90%, respectively, in one series) in patients with possible or suspected endocarditis.^82^^,^^88^

### Prosthetic valve emergencies

Patients with bioprosthetic valve dysfunction often present with subacute heart failure symptoms but may present abruptly with cardiogenic shock. A high index of suspicion for valve dysfunction is required for decompensating patients with a previous valve surgery and should be suspected in patients with muffled mechanical heart sounds in those with mechanical valves or newly increased valvular gradients seen on echocardiogram. The medical therapy of prosthetic valve dysfunction (eg, vasoactive medication or MCS selection) is similar to native valve stenotic or regurgitant pathologies. However, there are unique emergencies related to both bioprosthetic and mechanical valves that require special attention. Furthermore, there has been a shift from mechanical to less durable bioprosthetic valves resulting in the need for transcatheter options, as repeat valve surgery may carry significant morbidity and mortality.^89^, ^90^, ^91^

In the first 12 months, in particular the first 3 months,^10^ acute valve thrombosis should be considered for any decompensating patient with a history of valve surgery, especially those with mechanical valves (incidence 0.1% to 5.7% per patient-year).^92^ Bioprosthetic valve thrombosis usually occurs in the first 3 months after implantation.^92^, ^93^, ^94^ Diagnosis is generally made through TTE or TEE in the CICU and should prompt swift initiation of anticoagulation. Both North American and European guidelines give a Class I recommendation for repeat surgical intervention for critically ill patients with severe or obstructive thrombosis.^10^^,^^11^ For thrombosis of right-sided prostheses or for patients with prohibitive surgery risk, systemic fibrinolysis is recommended, although it carries a significant risk of complications, including thromboembolism (12%), major bleeding (5%), and recurrent thrombosis (11%).^95^ In a meta-analysis including 690 episodes of left-sided valve thrombosis, urgent surgery was associated with a statistically similar risk of mortality but a lower risk of thromboembolism, major bleeding, and recurrent thrombosis compared to fibrinolytic therapy.^96^ In a single arm study, a strategy of “low-dose, slow infusion” thrombolysis has been successful for patients with mechanical valve thrombosis but was associated with a higher risk of failure and more complications in patients with decompensated heart failure.^97^ Thrombolysis can be repeated and followed by serial echocardiograms to assess valve gradients and response to therapy.

Other etiologies of prosthetic valve decompensation include endocarditis (discussed previously), valve dehiscence, paravalvular leaks, and acute or chronic valvular dysfunction. Valve or ring dehiscence is a rare complication, often due to late infective endocarditis, which may present abruptly and require urgent surgical intervention. Classically, valve dehiscence is associated with a “rocking motion” (>15° in the mitral position) often with paravalvular leak on echocardiogram.^98^ Paravalvular leaks following surgical valve replacement vary by site and are often asymptomatic or have a benign course. However, 1% to 5% of patients can present with serious complications including hemolytic anemia and decompensated heart failure.^99^ Repeat valve surgery is often associated with significant morbidity and mortality and may not correct the original etiology causing the leak.^99^ More recently, plug implantation has been employed for paravalvular leak of both surgical and transcatheter valves with good effect.^100^

Bioprosthetic valve dysfunction results from pannus formation and deterioration of the leaflets or valve structures, ultimately resulting in valve regurgitation, stenosis, or both. Surgery remains the treatment of choice for appropriate candidates but is often limited by peri-operative risk in the decompensated, comorbid CICU patient. Successful percutaneous balloon valvuloplasty has been reported for both aortic and mitral stenotic bioprosthetic valves for patients with prohibitive surgical risk,^101^^,^^102^ but should be used with caution. Of the 5,006 patients with cardiogenic shock undergoing TAVR from the STS/ACC transcatheter valve therapy Registry study, 12% (n = 601) underwent valve-in-valve implantation and had similar outcomes to those with native valves.^38^ A review of 212 redo-TAVRs, 22% of which had NYHA functional class IV heart failure, from 37 countries found that 30-day survival was >95%. The rate of complications was low except for the requirement of new permanent pacemaker placement in approximately 10% of patients.^103^ Mitral valve-in-valve and valve-in-ring are technically more challenging, especially for critically ill patients with cardiogenic shock but have been reported with variable success.^104^

### Respiratory failure

**Figure 6:**
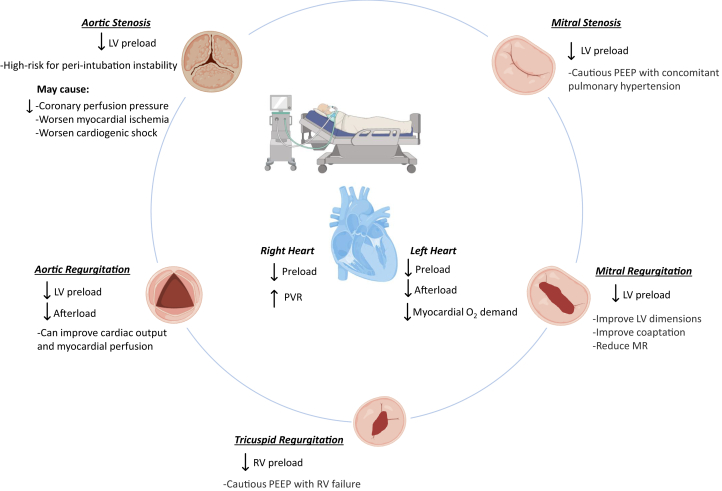
**Impact of Positive Pressure Ventilation on Valvular Heart Disease** Created in BioRender (2024). PEEP = positive end-expiratory pressure; PVR = pulmonary vascular resistance; RV = right ventricle; other abbreviations as in Figures 2 and 4.

Acute respiratory failure occurs in >30% of patients admitted to the CICU with severe valvular disease.^105^ Carefully managing noninvasive ventilation, endotracheal intubation, and liberation from mechanical ventilation are key aspects of the care of these patients. Indications for PPV in this population can include: 1) acute decompensation of valvular heart disease; 2) worsening heart failure compounded by valvular pathology; 3) noncardiac etiologies of respiratory failure in patients with severe valvular heart disease; 4) elective preoperative or preprocedural intubation for cardiac and noncardiac procedures, and v) prolonged mechanical ventilation and weaning in patients with concomitant valvular heart disease (Figure 6).

PPV reduces RV output by decreasing venous return and increasing pulmonary vascular resistance.^55^ Oxygenation and positive end-expiratory pressure (PEEP) may help alleviate hypoxia-driven pulmonary vasoconstriction to offset these physiological effects.^106^ In contrast, PPV may reduce LV preload, afterload, and myocardial oxygen demand.^55^ The net effect of PPV on patients with valvular disease depends on the complex interplay between the severity and type of valvular lesion, and the opposing effects on LV and RV function, preload, and filling pressures.

Initial therapy with noninvasive PPV, either continuous positive airway pressure or bilevel positive airway pressure, is reasonable to improve work of breathing and allow time for decongestion, improve hemodynamics, and hopefully prevent intubation. Appropriate intravenous access and arterial hemodynamic monitoring are usually important for management of such patients. For patients failing noninvasive PPV strategies, intubation should not be delayed but requires careful preparation. The intubating team should be made aware of any hemodynamically significant valvular disease. It is prudent to prepare vasoactive support and anticipate hypotension.

The use of induction medications and neuromuscular blockade may also have considerable hemodynamic effects on patient with cardiovascular dysfunction.^107^ In particular, patients with severe or critical AS are highly dependent on LV preload to maintain adequate cardiac output. PPV and vasodilating induction agents alone or in combination may lead to the acute reduction in LV preload and increase risk for peri-intubation hemodynamic instability. In the setting of severe AS, hypotension can lead to low coronary perfusion pressure and ensuing myocardial ischemia that may precipitate worsening cardiogenic shock. Specific induction medications such as propofol may be poorly tolerated in severe AS due to vasodilatory properties. Thus, induction agents with favorable profiles that minimize hypotension, such as etomidate or ketamine, are often preferred.^55^

After successful intubation, PEEP may be beneficial in patients with aortic and mitral regurgitation by reducing LV afterload. In 50 patients with severe MR undergoing MitraClip, notably not in cardiogenic shock, PEEP was found to improve LV dimensions, improve coaptation, and significantly reduce MR.^108^ However, significant MR may make liberation from mechanical ventilation challenging. Withdrawal of PPV may fail by acutely worsening MR and pulmonary edema.^109^ Decongestion prior to weaning PPV, afterload reduction during withdrawal of PPV, and extubation to immediate noninvasive PPV should be considered.

## Conclusions

Decompensated valvular disease is a common etiology and complicating factor in the management of critically ill patients admitted to the CICU. Medical therapy serves to optimize and stabilize patients in order to facilitate imaging and planning for definitive treatment. Ideally, valve surgery addresses the pathology and has historically been the definitive treatment. However, many patients are at prohibitive risk for valve surgery and evidence for transcatheter approaches in critically ill patients is increasing but remains limited. Future trials including critically ill patients that are dedicated to specific valvular pathologies are needed to improve the evidence base of this unique patient population. Accomplishing this goal will require various stakeholders, such as governmental bodies and industry, as well as several types of evidence generation, including dedicated registry, comparative effectiveness studies, and randomized controlled trials. Finally, due to the limited evidence and complex nature of decompensated valvular disease, a multidisciplinary approach is warranted.

## Funding support and author disclosures

Dr Rali has received consulting/speaking honorarium from Analog Devices, Abiomed, Caretaker Medical, Vectorious, Volumetrix, and Zoll. Dr Bhatt has received consulting fees for programs sponsored by Sanofi. Dr Grubb is a speaker, proctor, and principal investigator for Edwards Lifesciences; is a speaker, proctor, and advisory board member for Boston Scientific; is a speaker, proctor, principal investigator, advisory board member, and national principal investigator for Medtronic; and her employer has research contracts for clinical investigation of transcatheter aortic, mitral, and tricuspid devices from Edwards Lifesciences, Abbott Vascular, Medtronic, and Boston Scientific. Dr Morrow has received research grant support to TIMI Study Group through Brigham and Women’s Hospital from Abbott Laboratories, Abiomed, Amgen, Anthos Therapeutics, Arca Biopharma, AstraZeneca, Daiichi-Sankyo, Intarcia, Janssen, Merck, Novartis, Pfizer, Poxel, Quark Pharmaceuticals, Regeneron, Roche, Siemens, and Zora Biosciences; and consulting fees from Abbott Laboratories, Arca Biopharma, InCarda, Inflammatix, Merck, Novartis, and Roche Diagnostics. All other authors have reported that they have no relationships relevant to the contents of this paper to disclose.
